# Dry etching of ternary metal carbide TiAlC via surface modification using floating wire-assisted vapor plasma

**DOI:** 10.1038/s41598-022-24949-1

**Published:** 2022-11-27

**Authors:** Thi-Thuy-Nga Nguyen, Kazunori Shinoda, Hirotaka Hamamura, Kenji Maeda, Kenetsu Yokogawa, Masaru Izawa, Kenji Ishikawa, Masaru Hori

**Affiliations:** 1 Nagoya University, Nagoya, 464-8601 Japan; 2 Hitachi, Ltd., Tokyo, 185-8601 Japan; 3 Hitachi High-Tech Corp., Yamaguchi, 744-0002 Japan

**Keywords:** Materials for devices, Electronic devices, Surface chemistry

## Abstract

Dry etching of ternary metal carbides TiAlC has been first developed by transferring from wet etching to dry etching using a floating wire (FW)-assisted Ar/ammonium hydroxide vapor plasma. FW-assisted non-halogen vapor plasma generated at medium pressure can produce high-density reactive radicals (NH, H, and OH) for TiAlC surface modifications such as hydrogenation and methylamination. A proposed mechanism for dry etching of TiAlC is considered with the formation of the volatile products from the modified layer.

## Introduction

In a fin-type or nanosheet field effect transistor (FET) of a logic semiconductor device, it has been proposed to use metal gate materials, for examples, metal carbides (TiC, TiAlC) and metal nitrides (TiN, TaN, AlN, TiAlN)^1–5^. Ternary metal compound such as TiAlC belongs to high-melting point, high-hardness, and high-wear resistance materials^1,6,7^. Conventionally, the TiAlC film and TiC film in semiconductor devices are etched by wet etching using H_2_O_2_ mixtures^5,8–10^. However, a poor metal removability in wet etching requires a prolonged etching time to fully remove the target metals, and in the worst case, the metal gate can be damaged^5^. In order to fabricate the next generation FETs in semiconductor industries, it is strongly demanded to develop an etching method that enables to control the selective and isotropic removal of metal carbides (TiAlC, TiC, and AlC) at an atomic layer level^5,9,10^. No dry etching (plasma etching) of ternary material TiAlC with atomic level control has been developed yet.

Atmospheric pressure plasma (APP) and medium-pressure plasma techniques with a large difference in chemical kinetics compared to low-pressure plasma are able to miniaturize equipment size, fabrication cost, and energy consumption^11–17^. Medium-pressure plasma (0.2–50 kPa) can produce higher plasma density compared to vacuum plasma and larger plasma volume compared to APP^18–20^. In order to improve the plasma density at a remote region where the substrate is placed, we have inserted a long floating metal wire inside the discharge tube to enhance the electric field not only near the copper coil region, but also at a remote region. Therefore, a rich radical source (10^14^ cm^−3^) near the sample surface that is far from the coil region can be obtained^15^. The generated rich radical source can produce a large amount of etchant or co-reactant species to enhance the reaction rate with sample surface. This radical-rich environment plays an important role in controlling isotropic etching of 3D multilayer semiconductor devices.

Here we have first developed a new dry etching method for metal carbides such as ternary material TiAlC by using a floating wire (FW)-assisted vapor plasma of Ar gas mixed with vapor sources of NH_4_OH-based mixtures at medium pressure. Although the mechanism of wet etching and dry etching can be different because the formed compounds are dissolved in solutions in wet etching, and for plasma etching, the formed compounds should be volatile in the gas phase, the wet etching brings lots of useful ideas to develop new etching chemistries for traditional materials or dry etching methods for new materials. In this study, we aim to develop a new etching method (wet-dry etching or wet-like plasma etching) that can combine the advantages of wet etching (high isotropy and selectivity) and dry etching (high controllability) for new materials or hard-to-etch materials. Surface reaction plays a key role in developing atomic layer etching (ALE) processes, which normally are proceeded in multi-steps including surface modification to reduce the surface energy of sample surface in the first step, and then removal of modified layer in the next step. Here the surface modification, such as hydrogenation and methylamination, of the TiAlC film, was obtained by controlling the active radicals, such as NH, H, and OH. The treated TiAlC surface can be removed via the formation of modifed layers. Lastly, the mechanism for plasma etching of metal carbides (TiAlC, TiC, AlC) is proposed. The new etching method will be explored for etching metals and metal compounds such as nitrides, carbides, and oxides to determine if the selectivity and isotropy commonly seen in wet etching will also occur in dry etching.

## Materials and methods

### Sample preparation

**Figure 1:**
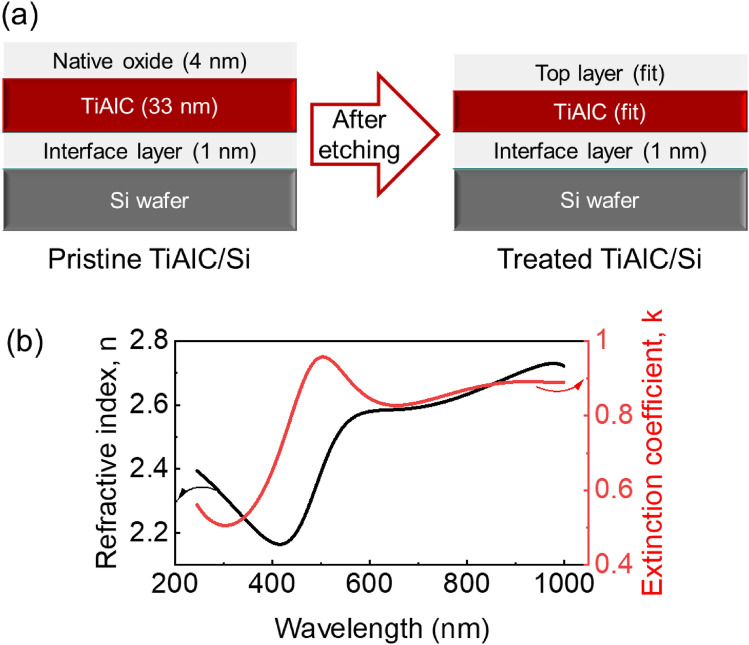
(**a**) A model for spectral fitting of ellipsometric data. A stratified layer model is constructed by a structure of TiAlC sample including top layer (native oxide, deposited layer, or modified layer), TiAlC layer, interface layer, and Si wafer. (**b**) Dispersions of refractive index and extinction coefficient of TiAlC film as functions of wavelength obtained by the Gen-Osc model with fitted parameters as listed in Table 1.

TiAlC films were prepared on Si wafer by vacuum evaporation with the Ti, Al sources, and C_2_H_2_ gas. TiAlC/Si samples were prepared with the size of 15 mm × 15 mm (for wet etching) and 15 mm × 20 mm (for dry etching). TiAlC films were analyzed by using an ellipsometry (M-2000, J.A. Woolam Co.) with a Xe arc light source (FLS-300). A model was used for spectral fitting of ellipsometric data of TiAlC sample including a top layer (native oxide, deposited layer, or modified layer), a TiAlC layer, an interface layer, and Si substrate, as shown in Fig. 1a. The pristine TiAlC film on Si substrate (TiAlC/Si) has a thickness of around 35 nm.

**Table 1:** Best-fit parameters of TiAlC layer obtained by Gen-Osc model.

	E_∞_ (eV)		
	2.257		

	Resistivity (Ω cm)^4^	Scattering time (fs)^4^	
Drude (RT)	0.00020278	0.504	

	Amplitude (eV)	Broadening (eV)	Energy (eV)
Lorentz 1	2.095593	0.9648	2.476
Lorentz 2	− 100.000000	0.8019	0.409
Lorentz 3	4.682310	8.1514	9.717

The dielectric function of the TiAlC film is expressed by the Gen-Osc model involving one Drude and three Lorentz oscillators^21^. The Gen-Osc model with the best-fit parameters is shown in Table 1.

Dispersions of optical constants including refractive index (n) and extinction coefficient (k) of TiAlC film as functions of the wavelength ranged from 245 to 1000 nm were obtained by the Gen-Osc model, as shown in Fig. 1b. The n and k values at 633 nm are 2.58 and 0.83, respectively. Cross-sectional microstructure and film thickness of samples were characterized by a cold field-emission scanning electron microscope (FE-SEM, SU-8230, Hitachi).

### Wet etching of TiAlC

To develop the new etching chemistries for TiAlC, both halogen-containing mixtures and non-halogen mixtures were used to test the potential etching chemistries. The 35 nm-TiAlC/Si samples (15 mm × 15 mm) were immersed in liquid mixtures such as peroxide solution that is a mixture of a 30% by weight water-based solution of hydrogen peroxide H_2_O_2_ (H_2_O_2_, 30 wt%), hydrochloric acid solution (HCl, 36 wt%), ammonium hydroxide solution (NH_4_OH, 29 wt%), and deionized water H_2_O. Table 2 shows a list of four experimental conditions (L1, L2, L3, and L4) of liquid mixtures for wet etching of TiAlC film.

**Table 2:** A list of four experimental conditions (L1, L2, L3, and L4) of liquid mixtures that were mixed at room temperature for wet etching of TiAlC film.

Condition	Liquid mixture	Volume ratio	Etch time (min)
**Halogen-containing mixtures**			
L1	HCl/H_2_O_2_/H_2_O	1:1:6	10
L2	HCl/H_2_O_2_	10:1	10
**Non-halogen mixtures**			
L3	H_2_O_2_	–	5
L4	NH_4_OH/H_2_O_2_/H_2_O	2.2:3:52	10

### Dry etching of TiAlC by FW-assisted vapor plasma

A dry etching method of TiAlC was developed by using FW-assisted vapor plasma. A long floating metal wire was placed inside the discharge tube to enhance the plasma density, that provides a rich radical source at a remote region^14,15^. The FW-assisted plasma can generate high densities of radicals, electronically excited particles, and photons in the visible and UV range, in which radicals are able to travel long distances. The charged particles from atmospheric-pressure plasmas have very short lifetime and are difficult to reach the substrate surface at a far distance^12,22^. In order to improve this, the FW-plasma is designed to assist the short-lived particles to reach the substrate surface placed far away from the plasma source.

**Figure 2:**
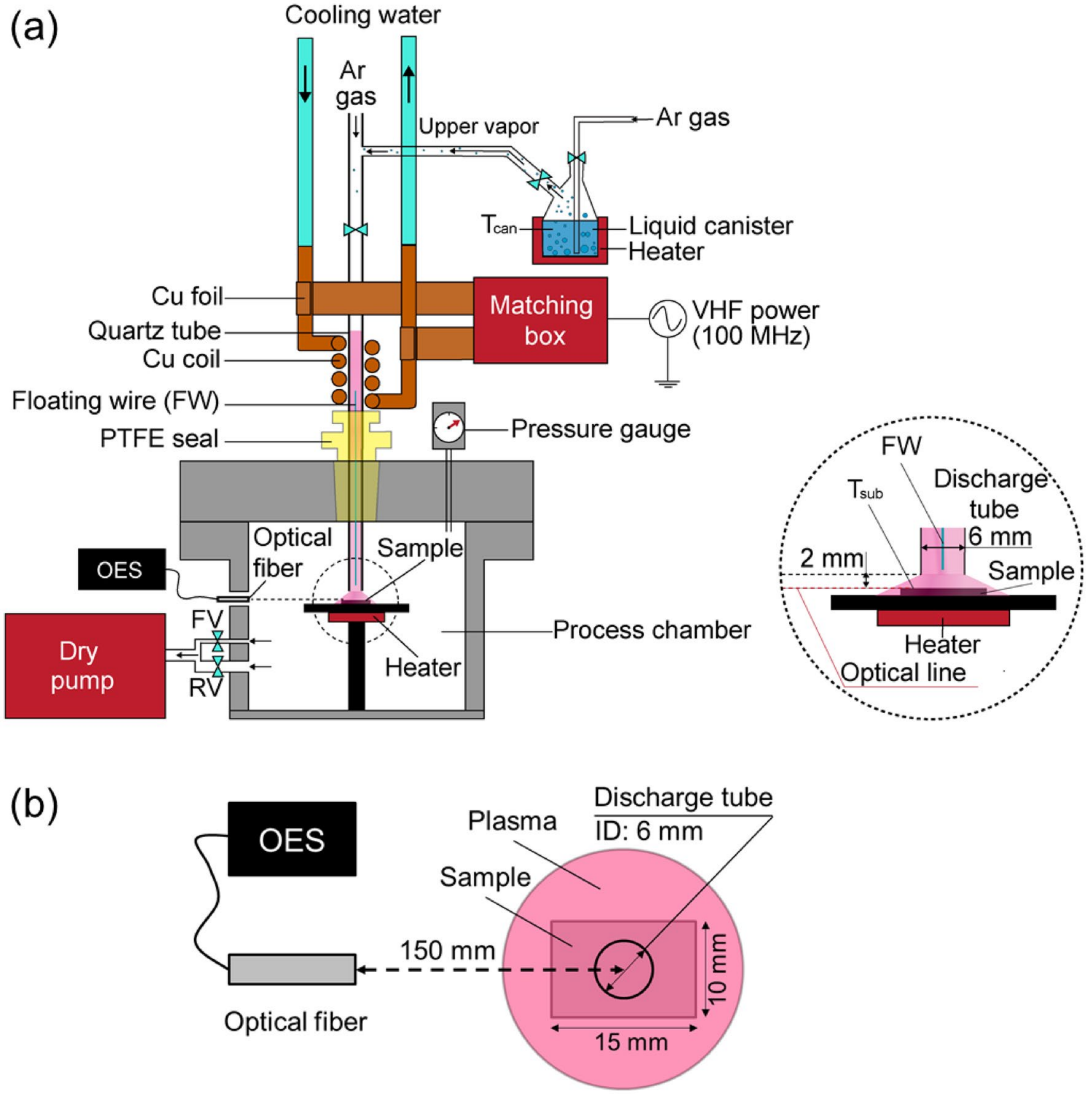
(**a**) Schematic structure of FW-plasma system. Vapors that can be introduced from upstream region. (**b**) Top view of measurement setup for OES of FW-plasma exposing to the sample.

The FW-assisted plasma was connected with a process chamber, a vacuum dry pump, and a heating unit, as shown in Fig. 2a. The FW-assisted plasma consists of a 500-mm-high discharge quartz tube (inner diameter of 6 mm) with a three-turn Cu coil connected with a very high-frequency (VHF) power of 100 MHz. A polytetrafluoroethylene (PTFE) seal was used to connect the discharge tube with the chamber. The FW made of metal wire is coated by a protective material to avoid the chemical reaction with plasma species. The distance between the center of copper coil and the sample surface is 140 mm. The distance between the sample surface and the discharge tube is 2 mm. Working pressure can be controlled from atmospheric pressure to medium pressure by using a rough valve (RV) and a fine valve (FV) between a dry pump (Kashiyama, NeoDry 15E) and the process chamber. The value of working pressure was recorded by a pressure gauge (Baratron MKS, 628B). In this study, the working pressure was controlled at 0.64 kPa.

The vapor was flowed from upstream with Ar gas to generate remote FW-Ar/upstream vapor plasma along the discharge tube. Liquid mixtures including deionized water H_2_O and two ammonium hydroxide solutions including (NH_4_OH, 28 wt%) and (NH_4_OH, 17 wt%) were prepared.

In order to generate FW-Ar/vapor plasma, the Ar gas of 1.5 standard liter per minute (slm) was used. The saturated vapor pressures of 100%, 28%, and 17% ammonia hydroxide at 25 °C are 1007 kPa, 83 kPa, and 30 kPa, respectively^23^, that are higher than the working pressure of 0.64 kPa used in this study. A list of three experimental conditions (P1, P2, and P3) to generate FW-Ar/vapor plasmas by various liquid mixtures injected from upper vapor line is shown in Table 3. The FW-Ar/vapor plasmas were generated at 100 W, and the temperature of the liquid canister (T_can_) was controlled at 70 °C. The substrate temperature (T_sub_) obtained from plasma discharge was measured by a thermocouple (around 150 °C, no additional heater was used).

**Table 3:** A list of three experimental conditions (P1, P2, and P3) to generate FW-Ar/vapor plasmas by various vapor mixtures injected from upstream region for TiAlC treatment.

Condition	Liquid mixture	T_can_ (°C)	Working pressure (kPa)	VHF power (W)	Exposure time (min)
P1	H_2_O	70	0.64	100	10
P2	NH_4_OH (17 wt%)	70	0.64	100	10
P3	NH_4_OH (28 wt%)	70	0.64	100	10

The gas flow of Ar gas is 1.5 slm.

### Plasma diagnostics

The optical emission spectra (OES) of the FW-Ar/vapor plasmas such as the emissions of Ar, OH, NH, H_β_, H_α_, O were detected by using a spectrometer (Ocean Photonics, HR4000CG-UV-NIR) with the wavelength from 200 to 900 nm. The measured point was set on the TiAlC film surface (Fig. 2b). The distance between the sample center and the head of optical fiber is 150 mm.

### Material characterization

In order to analyze the surface modification of the TiAlC surface, X-ray photoelectron spectra (XPS) were obtained using a spectrometer (ESCALAB 250; Vacuum Generator, UK) equipped with an Al *K*α (photon energy = 1486.6 eV) source in an analysis chamber evacuated to a base pressure of 5 × 10^−7^ Pa using an ion pump. Peak deconvolution and elemental concentrations were analyzed by the Advantage program. Depth profile of atomic concentration in an initial (pristine) TiAlC film deposited on Si substrate was evaluated with Ar sputtering at 3 keV and 1 µA to a sputter area of 2 mm × 2 mm for 10 min.

## Results

The initial (pristine) TiAlC film deposited on Si substrate was evaluated by depth profile of atomic concentration, as shown in Fig. S1. After removing the native oxide (Al–O, Ti–O, C=O) around 40% oxygen atomic concentration, the ratio of Ti:Al:C:O is around 28:22:40:10. Oxygen also exists inside the TiAlC film around 10%. The oxygen concentration increases to 20% at the interface between TiAlC film and Si substrate surface, that was assigned for the Si–O–C bond.

### Wet etching of TiAlC

**Figure 3:**
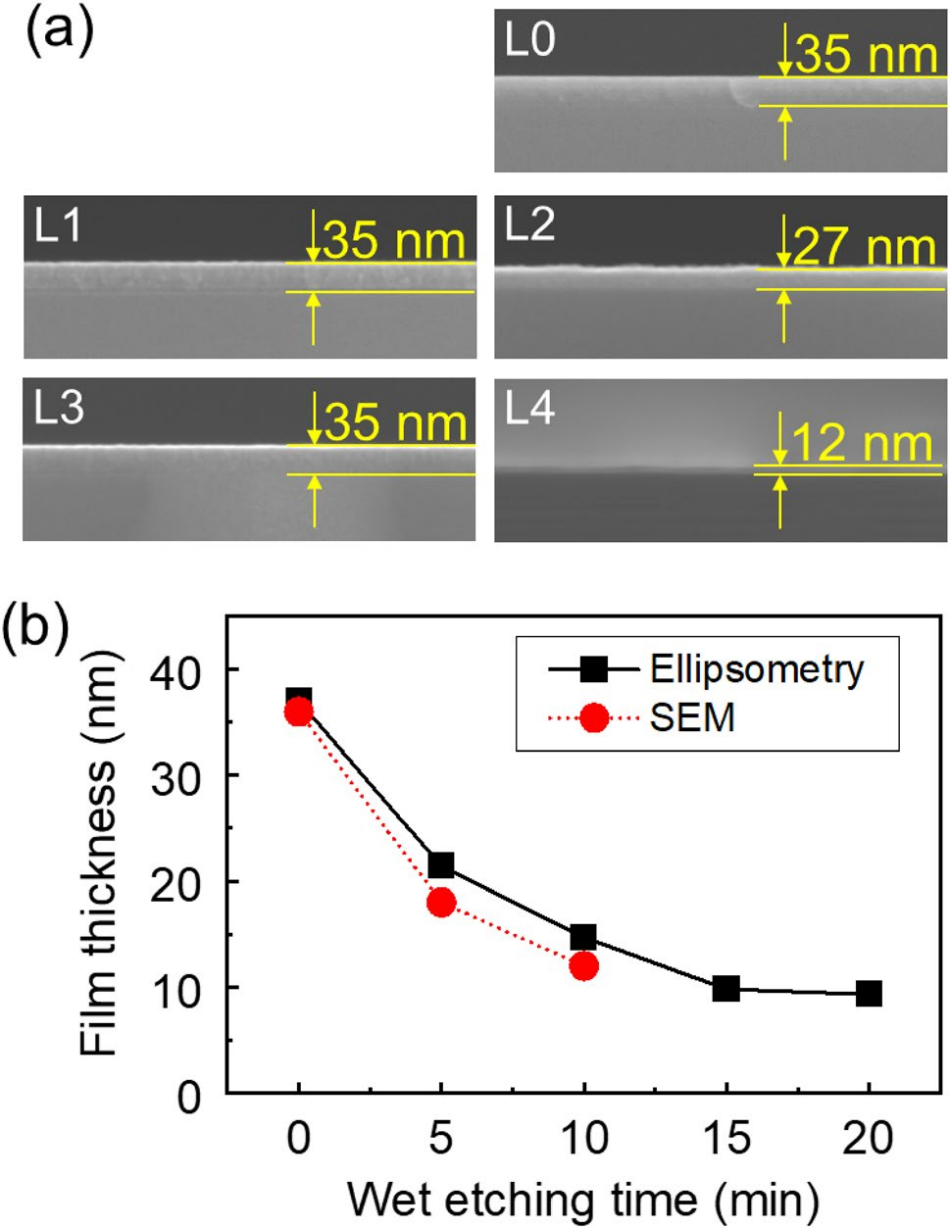
(**a**) SEM images of (L0) pristine TiAlC/Si sample and TiAlC/Si samples after wet etching in various solutions, (L1) HCl/H_2_O_2_/H_2_O (1:1:6) for 10 min, (L2) HCl/H_2_O_2_ (10:1) for 10 min, (L3) 30% H_2_O_2_ for 5 min, and (L4) NH_4_OH/H_2_O_2_/H_2_O (2.2:3:52) for 10 min. (**b**) Thickness of TiAlC film as a function of wet etching time in NH_4_OH/H_2_O_2_/H_2_O mixture. Film thickness was evaluated by using both ellipsometry and scanning electron microscopy.

In order to develop the etching chemistry for new materials, wet etching of TiAlC was conducted with different liquid mixtures including chlorine-based solutions and non-chlorine solutions. Figure 3a is a cross-sectional SEM images of TiAlC/Si samples before wet etching (35 nm-TiAlC, L0) and after wet etching by chlorine-based solutions and non-chlorine solutions. For chlorine-based solutions, no etching occurred with the mixture of HCl, H_2_O_2_, and H_2_O at a mixture ratio of 1:1:6 (condition L1). A low etch rate of 0.8 nm/min can be obtained with a mixture of HCl and H_2_O_2_ (10:1, condition L2).

For non-chlorine solutions, no etching occurred with H_2_O_2_ solution (condition L3). A high etch rate of 2.3 nm/min can be obtained by using a NH_4_OH/H_2_O_2_/H_2_O mixture (2.2:3:52, condition L4).

Wet etching of TiAlC by non-halogen liquid mixtures at different etch time was done by NH_4_OH/H_2_O_2_/H_2_O mixture. The surface modification of TiAlC film after wet chemical etching in NH_4_OH/H_2_O_2_/H_2_O mixture at room temperature is analyzed by XPS spectra, as shown Fig. S2. The intensity of Ti 2p and Al 2p was significantly reduced after 5 min and 10 min etching, whereas C–C bond at 284.8 eV (C 1s) was significantly increased. N–H and C–N peaks (around 400 eV) can be found in N 1s spectra after etching. This indicates that the compounds containing C–C and C–N bonds after wet etching of TiAlC surface are not able to dissolve in NH_4_OH/H_2_O_2_/H_2_O mixture. As a result, Ti and Al could be dissolved in the solution; however, the TiAlC surface was covered with the C-C and C–N bonds.

Based on the XPS results and the studies of Kakihana et al. and Sirijaraensre et al. on dissolution of Ti compounds^24,25^, the reaction of NH_4_OH/H_2_O_2_/H_2_O mixture with TiAlC to form hydroxylamine or other compounds can be assumed as follows.

For Ti–Al bond:

1 $${\text{Ti}}{-}{\text{Al }} + {\text{ H}}_{{2}} {\text{O}}_{{2}} \to {\text{ Ti}}\left( {{\text{Al}}} \right){\text{-OH}} \cdots {\text{H}}_{{2}} {\text{O}}_{{2}} \to {\text{ Ti}}\left( {{\text{Al}}} \right){\text{-OOH}} \cdots {\text{H}}_{{2}} {\text{O,}}$$

2 $${\text{Ti}}\left( {{\text{Al-}}} \right){\text{OOH}} \cdots {\text{H}}_{{2}} {\text{O }} + {\text{ NH}}_{{3}} \to {\text{ Ti}}\left( {{\text{Al-}}} \right){\text{OOH}} \cdots {\text{NH}}_{{3}} + {\text{ H}}_{{2}} {\text{O}},$$

3 $${\text{Ti}}\left( {{\text{Al}}} \right){\text{-OOH}} \cdots {\text{NH}}_{{3}} \to {\text{ Ti}}\left( {{\text{Al}}} \right){\text{-OH}} \cdots {\text{ONH}}_{{3}} \to {\text{ Ti}}\left( {{\text{Al}}} \right){\text{-OH}} \cdots {\text{NH}}_{{2}} {\text{OH}}.$$

For Ti–C or Al–C bond:

4 $${\text{Ti}}\left( {{\text{Al}}} \right){-}{\text{C}} + {\text{ H}}_{{2}} {\text{O}}_{{2}} \to {\text{ Ti}}\left( {{\text{Al}}} \right){-}{\text{C}}{-}{\text{OH}} \cdots {\text{H}}_{{2}} {\text{O}}_{{2}} \to {\text{ Ti}}\left( {{\text{Al}}} \right){-}{\text{C}}( = {\text{O}}){\text{OH}} \cdots {\text{H}}_{{2}} {\text{O}},$$

5 $${\text{Ti(Al)C(}}={\text{O)OH}} \ldots {\text{H}}_{2}{\text{O}} + {\text{NH}}_{3} \to {\text{Ti(Al)}}-{\text{C}}(={\text{O}}){\text{OH}} \ldots {\text{NH}}_{2}{\text{OH}}$$

6 $${\text{n}}\left[ {{\text{Ti}}\left( {{\text{Al}}} \right) - {\text{C}}} \right] \, + {\text{ nNH}}_{{3}} + {\text{ nH}}_{{2}} {\text{O}}_{{2}} \to {\text{ nTi}}\left( {{\text{Al}}} \right) - {\text{OH}} \cdots {\text{NH}}_{{2}} {\text{OH}} + {\text{ C}}_{{\text{n}}} {\text{H}}_{{{\text{2n}} + {1}}} - {\text{NH}}_{{\text{x}}} .$$

These hydrogen bonding structures, such as Ti(Al)-OH⋯NH_2_OH and Ti(Al)-COOH⋯NH_2_OH, are soluble in water. However, the structures (such as C_n_C_2n+1_–NH_x_), having C–C and C–N, are insoluble in H_2_O, this forms a barrier to stop wet etching.

Table S1 shows the film thickness of TiAlC film and the surface layer (top layer) etched by the liquid mixture of NH_4_OH, H_2_O_2_, and H_2_O (condition L4). The film thickness reduces with an increase of etch time. The etch rate decreased from 4.9 to 2.1 nm/min when the etch time was increased from 5 to 15 min. The etch rate reduces owing to a C layer formed on TiAlC surface, this becomes a barrier for the reaction between the liquid and TiAlC surface.

Figure 3b presents the thickness of TiAlC films as a function of etch time in the NH_4_OH/H_2_O_2_/H_2_O mixture. The etch rate decreases from 4.9 to 2.1 nm/min when the etch time is increased from 5 to 15 min due to a C layer forming on the TiAlC surface. The etch stop at the Si–O–C interface between the TiAlC film and the Si substrate. Therefore, without removal of the C layer, it is difficult to control the etch rate of TiAlC in a NH_4_OH/H_2_O_2_/H_2_O mixture.

Wet chemical etching of TiAlC film brings potential chemistries for the development of dry etching of TiAlC film. In addition to chlorine-based etching, non-chlorine etching with elements such as H, N, O or their combinations such as NH_x_, OH, NO_x_ can be candidates for reactive species in plasma etching of TiAlC.

### Dry etching of TiAlC by a remote FW-assisted vapor plasma

With considerations about the volatile products for etching metal compounds, especially for the ternary or more than three-element compounds such as TiAlC, the potential plasma etchants are halogen-based etchants. Fluorine-based plasma forms AlF_3_ that is a non-volatile product (boiling point (b.p.) more than 1290 °C)^26,27^. Chlorine, bromide, or iodine-based plasmas can form volatile products, such as TiCl_4_ (b.p. ~ 136 °C) and AlCl_3_ (b.p. ~ 183 °C)^27,28^. In order to obtain high selective removal between Ti compounds, halogen-based plasma is limited to use due to formation of the same volatile product such as TiCl_4_.

The wet etching of non-halogen mixture of ammonium hydroxide solution, peroxide solution, and deionized water shows promised results with higher etch rate than the halogen-based mixture. This wet chemical etching of TiAlC film brings potential chemistries for the development of non-halogen dry etching of TiAlC film with etchants based on elements such as H, N, and O or their combinations to produce reactive species in plasma etching of TiAlC such as NH_x_, H, OH, or NO_x_. In this study, vapors were prepared based on the liquid mixtures that were used in wet etching. These vapors were used for generating reactive species in plasma etching. The remote FW-Ar/vapor plasma can generate various radicals by using different liquid mixtures, this can be used to for selective chemical reactions with TiAlC surface.

Vapor was flowed from upstream region with Ar gas to generate the remote FW-Ar/vapor plasma along the discharge tube. The vapors were prepared without using H_2_O_2_. The mixtures of Ar gas and H_2_O vapor, Ar gas and NH_4_OH 17% vapor, and Ar gas and NH_4_OH 28% vapor respectively generate Ar/H_2_O plasma (condition P1), Ar/NH_4_OH-17 plasma (condition P2), and Ar/NH_4_OH-28 plasma (condition P3) (Table 3).

**Figure 4:**
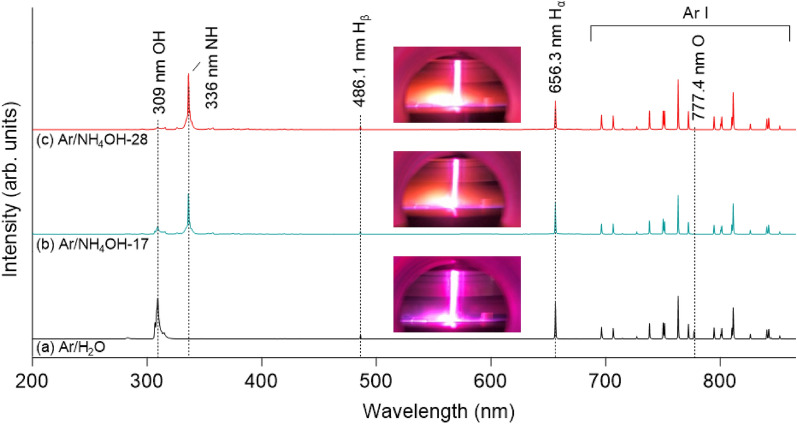
OES of plasmas generated with (**a**) Ar/H_2_O plasma, (**b**) Ar/NH_4_OH-17 plasma, and (**c**) Ar/NH_4_OH-28 plasma at 100 W and 0.64 kPa.

Figure 4 presents the OES of Ar/H_2_O plasma and Ar/NH_4_OH plasmas at 100 W and 0.64 kPa. Various plasma colors with different vapor mixtures can be observed from the insets of photograph images. H_β_ (486.1 nm) emission is detected in all spectra, whereas no O emission line (777.4 nm) can be seen from Ar/NH_4_OH-28 plasma. Strong emission lines for OH, NH, and H_α_ compared with Ar emission are detected. Intensity ratio of OH emission over NH emission can be controlled by different liquid mixtures. In case of using the Ar/NH_4_OH plasmas, mainly NH_3_ from NH_4_OH solution was injected to the chamber due to lower boiling point of NH_3_ (− 33 °C) compared to H_2_O (100 °C) at 1 atm^29^. NH_3_ can dissociate by energetic electron collisions to form radicals such as NH_2_ (571 nm), NH (336 nm), N_2_ (357 nm), and H_β_ (486.1 nm), and H_α_ (656.3 nm)^30–33^.

At the power of 100 W, inductive component is dominant (H-mode), and hence, high-density of the Ar/NH_4_OH plasma can be generated with mainly NH radical (NH*) and H radical (H*) as follows:

7 $${\text{NH}}_{{3}} + {\text{ e}}^{ - } \to {\text{ NH}}_{{2}} * \, + {\text{ H}}*$$

8 $${\text{ and NH}}_{{2}} * + {\text{ e}}^{ - } \to {\text{ NH}}* \, + {\text{H}}*.$$

The selective generation of reactive species such as OH, O, NH, H radicals can be controlled by using different liquid mixtures. Ar/H_2_O plasma mainly produces OH, H, and O radicals, whereas Ar/NH_4_OH plasma mainly produces NH and H radicals. Ar/NH_4_OH-17 plasma can generate all of species that were listed by both Ar/H_2_O plasma and Ar/NH_4_OH-28 plasma such as NH, H, OH, and O radicals. Depending on each application, the generation of selective radicals can be controlled to modify the surface of metal compounds such as oxidation, hydrogenation, nitridation, and methylamination. This modified layer can be removed by heating, ion bombardment, or is exchanged by other ligands for selective removal over other materials.

Film thickness of TiAlC was changed by all of the FW-Ar/vapor plasmas including Ar/H_2_O plasma (condition P1) and Ar/NH_4_OH plasmas (condition P2 and P3). Etching effect of TiAlC by FW-Ar/NH_4_OH plasma was evaluated by ellipsometry. In case of using Ar/H_2_O plasma for 10 min, the film thickness increases 1.61 nm due to oxidation of metal surface. In case of using Ar/NH_4_OH plasmas, the film thickness decreases around 1.70 nm after 10 min exposing for both Ar/NH_4_OH-17 plasma (1.76 nm) and Ar/NH_4_OH-28 plasma (1.67 nm), proving etching occurred with TiAlC surface when exposing TiAlC to FW-Ar/NH_4_OH plasma. There is no significant difference in etch rates between Ar/NH_4_OH-17 and Ar/NH_4_OH-28 because in addition to forming the volatile products, nitridation also occurred at higher concentration of ammonium hydroxide solution (Fig. 6) at longer treatment time. The experiment in Fig. 6 was done with only 10 min treatment, in which the nitridation was not occurred seriously, so the etch rate in both cases are almost the same. The results prove that radicals (NH, H) from Ar/NH_4_OH plasma can reacts with TiAlC surface to form of volatile products.

### Surface modification of TiAlC by the remote FW-assisted vapor plasma

**Figure 5:**
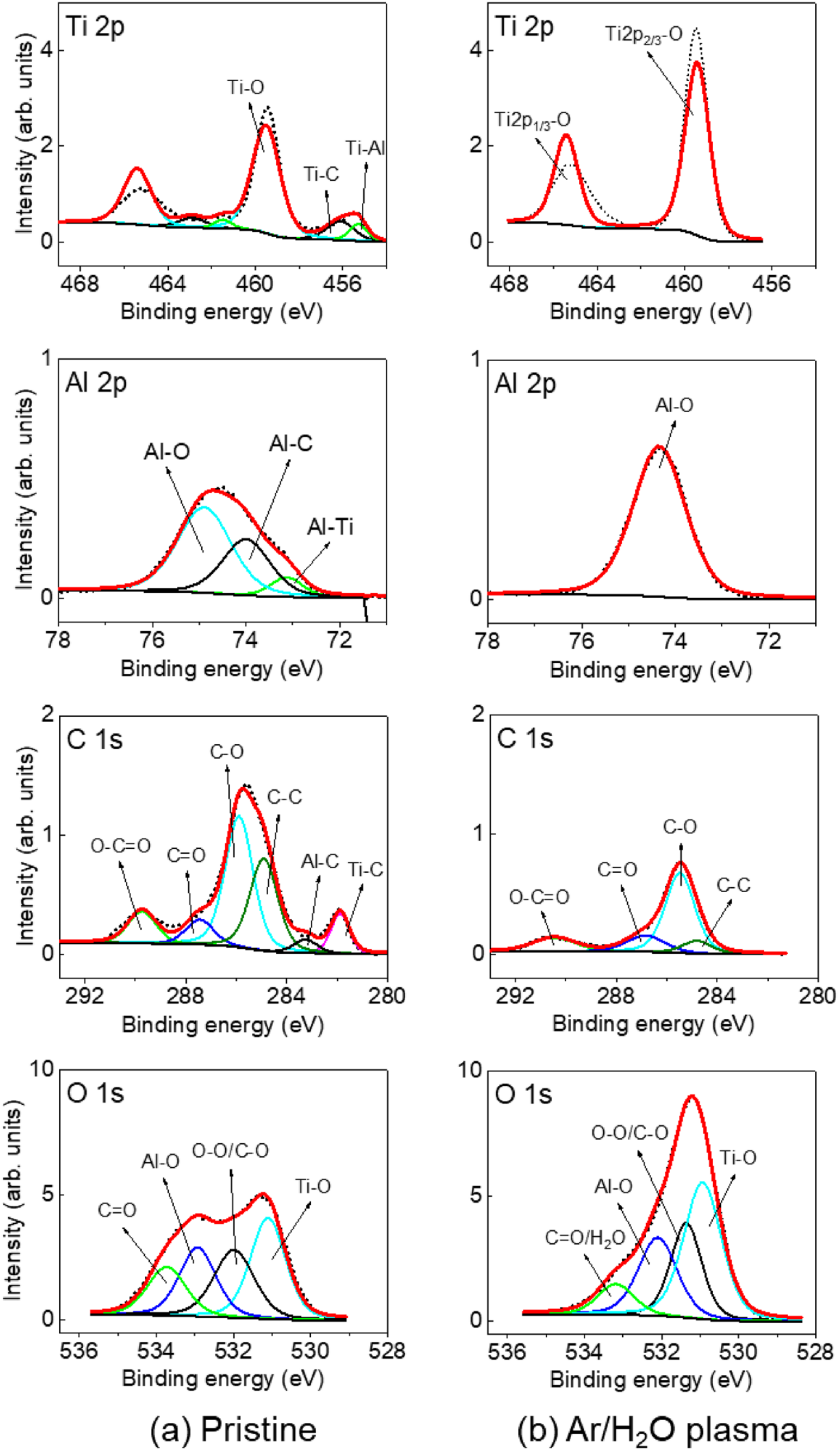
XPS spectra obtained on the surface of TiAlC film (**a**) before and (**b**) after exposure to Ar/H_2_O plasma at 100 W and 0.64 kPa.

The surface modification of TiAlC film before (pristine) and after exposure to Ar/H_2_O plasma at 100 W and 0.64 kPa was analyzed by XPS spectra, as shown in Fig. 5. In case of using Ar/H_2_O plasma (Fig. 5b), surface oxidation of TiAlC occurs obviously with the removal of Ti–C, Al–C bonds. Effect of water vapor in water-containing atmospheric-pressure plasma has been studied^34–38^. The Ar/H_2_O plasma jet was reported for polymer etching and surface modification at atmospheric pressure, in which OH radical plays a dominant role or is an effective etchant in polymer etching more than H radical and O radical^39,40^. In this study, the O atomic concentration on TiAlC surface increases from 41% (pristine) to 51% (Ar/H_2_O plasma). The FW-Ar/H_2_O plasma can produce very high density of OH and O radicals, and therefore, fully oxidation (may including hydroxylation) of TiAlC surface with only Ti–O(H) and Al–O(H) bonds were detected, and the removal of Ti–C and Al–C bonds were obtained. Only C from TiAlC compound can be etched by Ar/H_2_O plasma.

**Figure 6:**
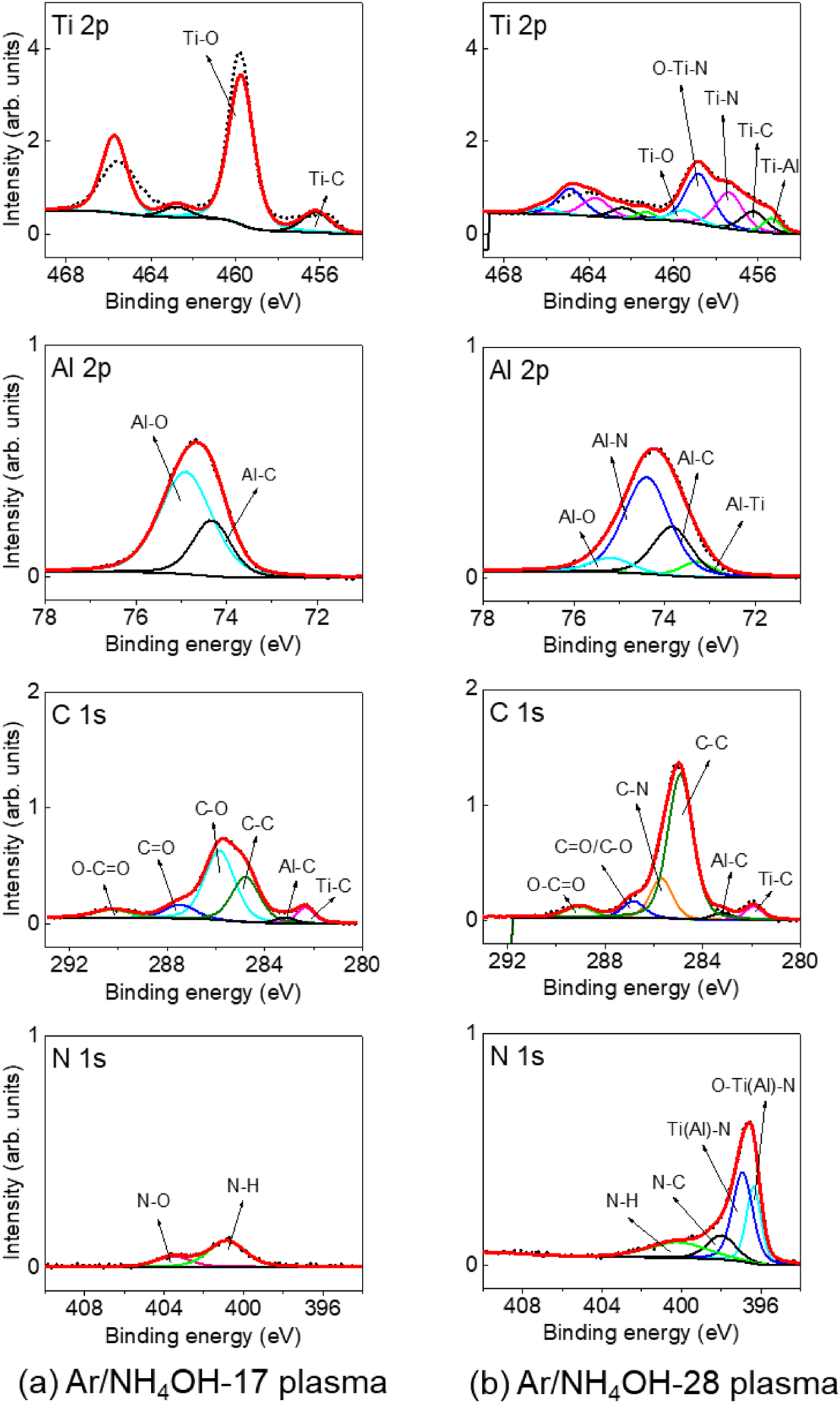
XPS spectra obtained on the surface of TiAlC film after exposure to (**a**) Ar/NH_4_OH-17 plasma and (**b**) Ar/NH_4_OH-28 plasma at 100 W and 0.64 kPa.

The surface modification of TiAlC film after exposure to Ar/NH_4_OH plasmas at 100 W and 0.64 kPa is shown in Fig. 6. Although etching depths of TiAlC samples after exposing to Ar/NH_4_OH plasmas for 10 min including Ar/NH_4_OH-17 plasma and Ar/NH_4_OH-28 plasma are almost the same, the surface modification of TiAlC for these two samples are quite different. In case of using Ar/NH_4_OH-17 plasma, NH and H radicals are more dominant compared to OH and O radicals (Fig. 4). A modest amount of N atom on TiAlC surface (less than 2%) can be detected in N 1s spectrum with the formation of N–H and N–O bonds (Fig. 6a). The present of a small amount of OH and O radicals play an important role in hindering nitridation of TiAlC surface. In contrast with the Ar/H_2_O plasma, the shape of C 1s of sample treated by the Ar/NH_4_OH plasma shows the same tendency with that of pristine sample, indicating that both Ti–C and Al–C bonds still exist on TiAlC surface. After exposing to Ar/NH_4_OH-17 plasma, etching occurred with the removal of volatile products, the surface of TiAlC became almost the same with that of pristine sample.

**Figure 7:**
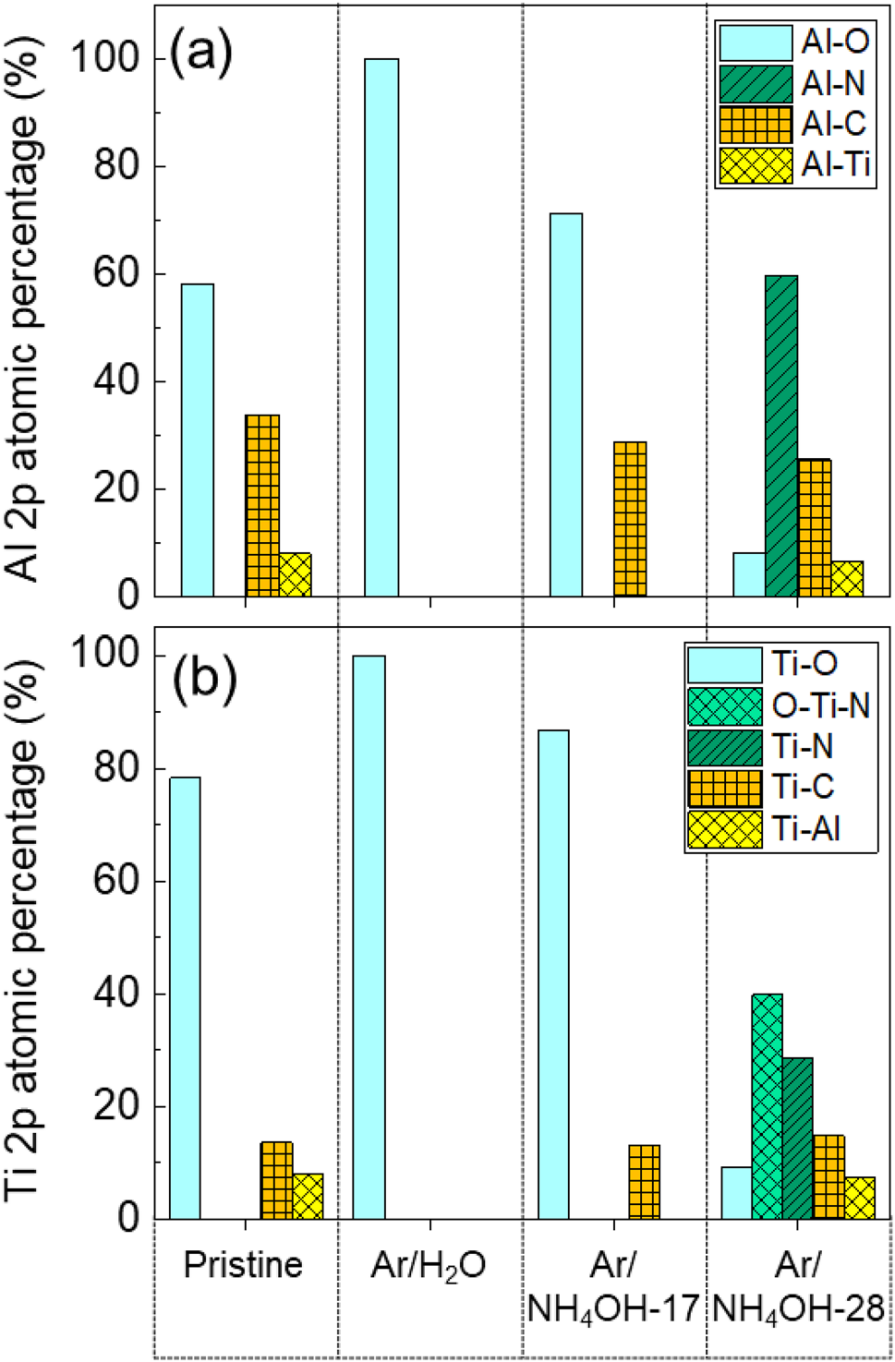
Chemical bond (Ti 2p and Al 2p) percentage of TiAlC surface before (pristine) and after exposure to Ar/H_2_O plasma and Ar/NH_4_OH plasmas at 100 W and 0.64 kPa.

In case of using Ar/NH_4_OH-28 plasma, nitridation and amination can be detected with a significant change in shape of Ti 2p peak and N 1s peak compared to using Ar/H_2_O plasma and Ar/NH_4_OH-17 plasma (Fig. 6b). Main species such as NH and H radicals were detected from OES result (Fig. 4). More N atoms are penetrated into TiAlC with 6.24% (Ar/NH_4_OH-28 plasma) compared to 1.83% (Ar/NH_4_OH-17 plasma). In N 1s spectrum, N–H, C–N, Ti–N, and O–Ti–N bonds can be respectively detected at 400.18 eV, 397.99 eV, 396.92 eV, and 396.36 eV. Both of the Ti–C and Al–C bonds of sample treated by the Ar/NH_4_OH plasmas are still remained (Fig. 7a,b), and Ti(Al)-O bond are removed and replaced by Ti(Al)–N. The remains of C in metal-C bonds are important for developing etching of metal carbides. This C in TiAlC can combine with N(H) and H from the Ar/NH_4_OH plasma to form Al–CH_3_, Ti(Al)–N–CH_3_, and Ti(Al)–O–C_n_H_2n+1_ bonds in volatile products, whereas it is impossible to form these bonds on TiN surface by the Ar/NH_4_OH plasma. The surface of TiAlC is very sensitive with oxidization, and the XPS measurement was conducts after exposure the samples in air, so the atomic concentration of O is over 40% in all cases.

The FW-Ar/vapor plasma has been developed to be used for dry etching of ternary TiAlC material. Surface modification is an indispensable step to approach the atomic layer etching.

## Discussion

### Surface modification and etching of TiAlC using FW-Ar/NH_4_OH plasma

The NH (NH*) and H (H*) radicals generated by FW-Ar/NH_4_OH plasma can modify the metal carbides (MC) to form volatile metal–nitrogen–hydrocarbons M(N(CH_3_)_2_)_n_ or metal-hydrocarbons M(CH_3_)_n_ via hydrogenation and methylamination of TiAlC surface. The formation of volatile product from TiAlC surface can be explained in the good agreement with Tamaki et al. in the formation of titanium nitride layer^32^. NH radicals and H radicals play a key role in this reaction. NH radicals and H radicals from plasma are first adsorbed on the TiAlC surface. The H radical can work as a catalyst to change the NH radical into an active N atom (N_act_) that can be absorbed into TiAlC surface to form Ti(Al)–N–CH_3_ bond and release H_2_ gas. In addition, numerous active H radicals (H_act_) can also penetrate to the sample surface for hydrogenation of TiAlC to form Al–CH_3_ group. Hydrogenation and methylamination of TiAlC surface can occur simultaneously in the presence of NH radicals (NH*) and H radicals (H*).

9 $${\text{H}}* \, + {\text{ NH}}* \, + {\text{ Ti}}\left( {{\text{Al}}} \right){\text{C }} \to {\text{ Ti}}\left( {{\text{Al}}} \right){\text{C }} + {\text{ N}}_{{{\text{act}}}} + {\text{ H}}_{{{\text{act}}}} + {\text{ H}}_{{2}} \to {\text{ Ti}}\left( {{\text{Al}}} \right) - {\text{N}}{-}{\text{CH}}_{{3}} + {\text{Al}}{-}{\text{CH}}_{{3}} + {\text{ Ti}}\left( {{\text{Al}}} \right){-}{\text{N }} + {\text{ H}}_{{2}} .$$

Small amount of OH from the plasma can hinder the nitridation by forming NO_x_ gas.

10 $${\text{H}}* \, + {\text{ NH}}* \, + {\text{ OH}}* \, + {\text{ Ti}}\left( {{\text{Al}}} \right){\text{C }} \to {\text{ Ti}}\left( {{\text{Al}}} \right){\text{C }} + {\text{ N}}_{{{\text{act}}}} + {\text{ H}}_{{{\text{act}}}} + {\text{ O }} + {\text{ H}}_{{2}} \to {\text{ Ti}}\left( {{\text{Al}}} \right){-}{\text{N}}{-}{\text{CH}}_{{3}} + {\text{ Al}}{-}{\text{CH}}_{{3}} + {\text{ N}}{-}{\text{O }} + {\text{ H}}_{{2}} .$$

Overall, the formation of bonds such as Al–CH_3_, Ti(Al)–N–CH_3_ or Ti(Al)–O–C_n_H_2n+1_ (in case of pristine sample is TiAlOC) shows a potential of producing volatile products such as Al(R or R′ or R′′)_3,_ and Ti(R or R′ or R′′)_4_, in which R is –CH_3_, R′ is –N–CH_3_, and R′′ is –O–C_n_H_2n+1_. This modified layer could be removed by forming volatile products.

### Proposed mechanism of dry etching TiAlC

**Figure 8:**
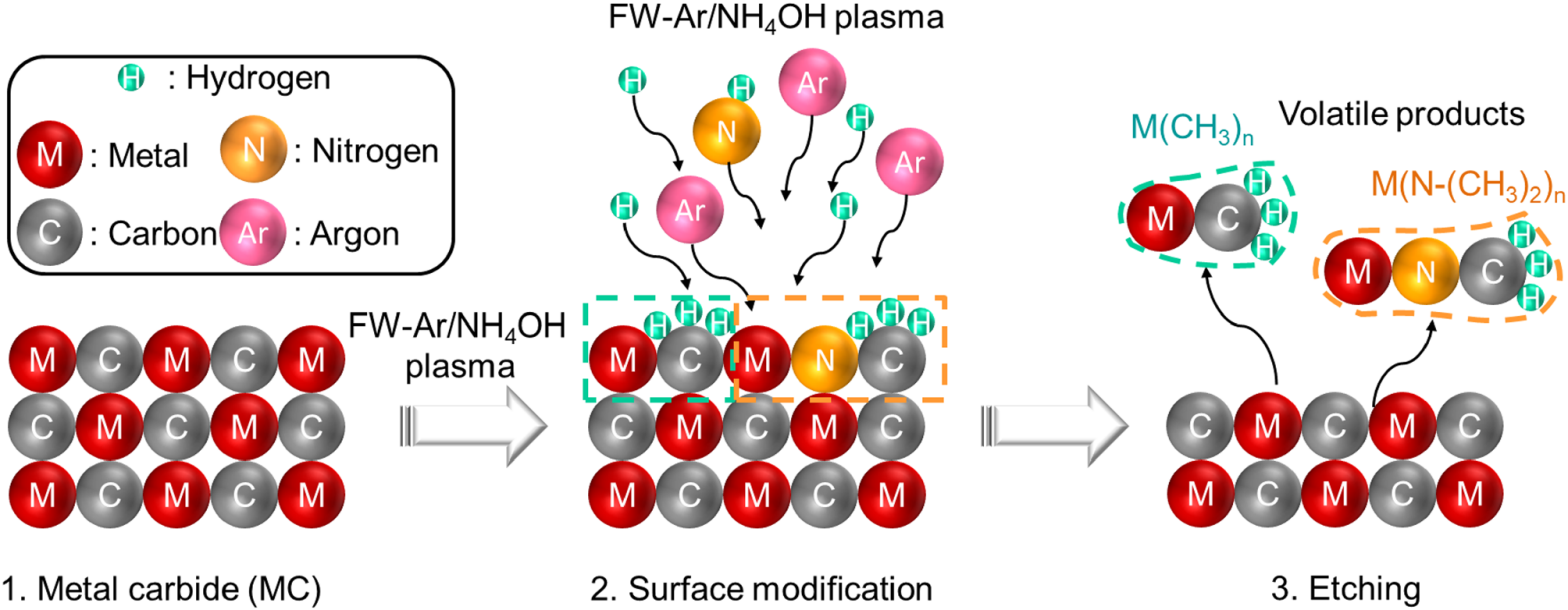
Proposed mechanism for plasma etching of metal carbide (MC) using FW-Ar/NH_4_OH plasma.

A plasma etching process for metal carbides MCs such as TiAlC, TiC, and AlC using FW-assisted plasma is demonstrated here (Fig. 8). The surface modification (hydrogenation, and amination) by reactive radicals (NH and H) and the removal of volatile metalorganic products, such as Al(CH_3_)_3_, dimer of Al(N(CH_3_)_2_)_3_, and Ti(N(CH_3_)_2_)_4_, are designed for plasma etching of metal carbides. The reactive radicals can be produced by ammonium hydroxide vapor plasma, NH_3_ plasma, H_2_ and NH_3_ mixture (H_2_/NH_3_) plasma, N_2_ and NH_3_ mixture (N_2_/NH_3_) plasma, or N_2_ and H_2_ mixture (N_2_/H_2_) plasma, or alcohol and ammonium hydroxide vapor mixture (C_n_H_2n+1_OH/NH_4_OH; n = 1–4) plasma. Surface modifications (hydrogenation and amination) of the TiAlC film were controlled by the active radicals produced from FW-assisted non-halogen plasma. The chemical bonds, involving (1) metal and methyl group (Al–CH_3_), (2) metal and dimethylamine group (Ti(Al)–N(CH_3_)_2_), and (3) metal and alkoxy group (Ti(Al)–OC_n_H_2n+1_) on TiAlC surface play an important role to form volatile products. Hence, the FW-assisted plasma, that is a rich radical source, is expected to be applied for atomic layer etching of metal and metal compounds in semiconductor device fabrication.

## Conclusions

A dry etching method for a ternary metal carbide TiAlC at atomic level has been developed here by transferring from wet etching to dry plasma etching using FW-assisted non-halogen vapor plasma of ammonium hydroxide. Surface modifications of the TiAlC film were controlled by exposing to the active radicals such as H, NH, and OH radicals, produced from the FW-assisted plasma. Mechanism for removal of metal carbide MC (TiAlC, TiC, AlC) is presented by using NH radicals and H radicals. This FW-assisted plasma technique is expected to be available for highly selective and isotropic atomic layer etching of metal and metal compounds in semiconductor device fabrication.

## Supplementary Information

Supplementary Information.

## Data Availability

The datasets generated during and/or analyzed during the current study are available from the corresponding author on reasonable request.
