# Supplementary material for: Dry etching of ternary metal carbide TiAlC via surface modification using floating wire-assisted vapor plasma
Source: Sci Rep. 2022 Nov 27;12:20394. doi: 10.1038/s41598-022-24949-1 (PMC9701795; doi:10.1038/s41598-022-24949-1)
Supplement: Supplementary file 1 — Supplementary Information. [file 41598_2022_24949_MOESM1_ESM.docx]

Supplementary Information

Dry etching of ternary metal carbide TiAlC via surface modification using floating wire-assisted vapor plasma

Thi-Thuy-Nga Nguyen,^1,*^ Kazunori Shinoda,^2^ Hirotaka Hamamura,^2^ Kenji Maeda,^3^ Kenetsu Yokogawa,^3^ Masaru Izawa,^3^ Kenji Ishikawa,^1,*^ and Masaru Hori^1^

^1^Nagoya University, Nagoya 464-8601, Japan

^2^Hitachi, Ltd., Tokyo 185-8601, Japan

^3^Hitachi High-Tech Corp., Yamaguchi 744-0002, Japan

^*^nguyen@plasma.engg.nagoya-u.ac.jp (ORCID 0000-0002-2170-2488)

ishikawa.kenji@nagoya-u.jp (ORCID 0000-0002-8288-6620)

Keywords: Metal carbide, TiAlC, Floating wire-assisted plasma, Dry etching, Plasma etching, Wet etching

|  |
| --- |

Figure S1. Depth profile of atomic concentration in TiAlC films evaluated by X-ray photoelectron spectroscopy with Ar sputtering.

| 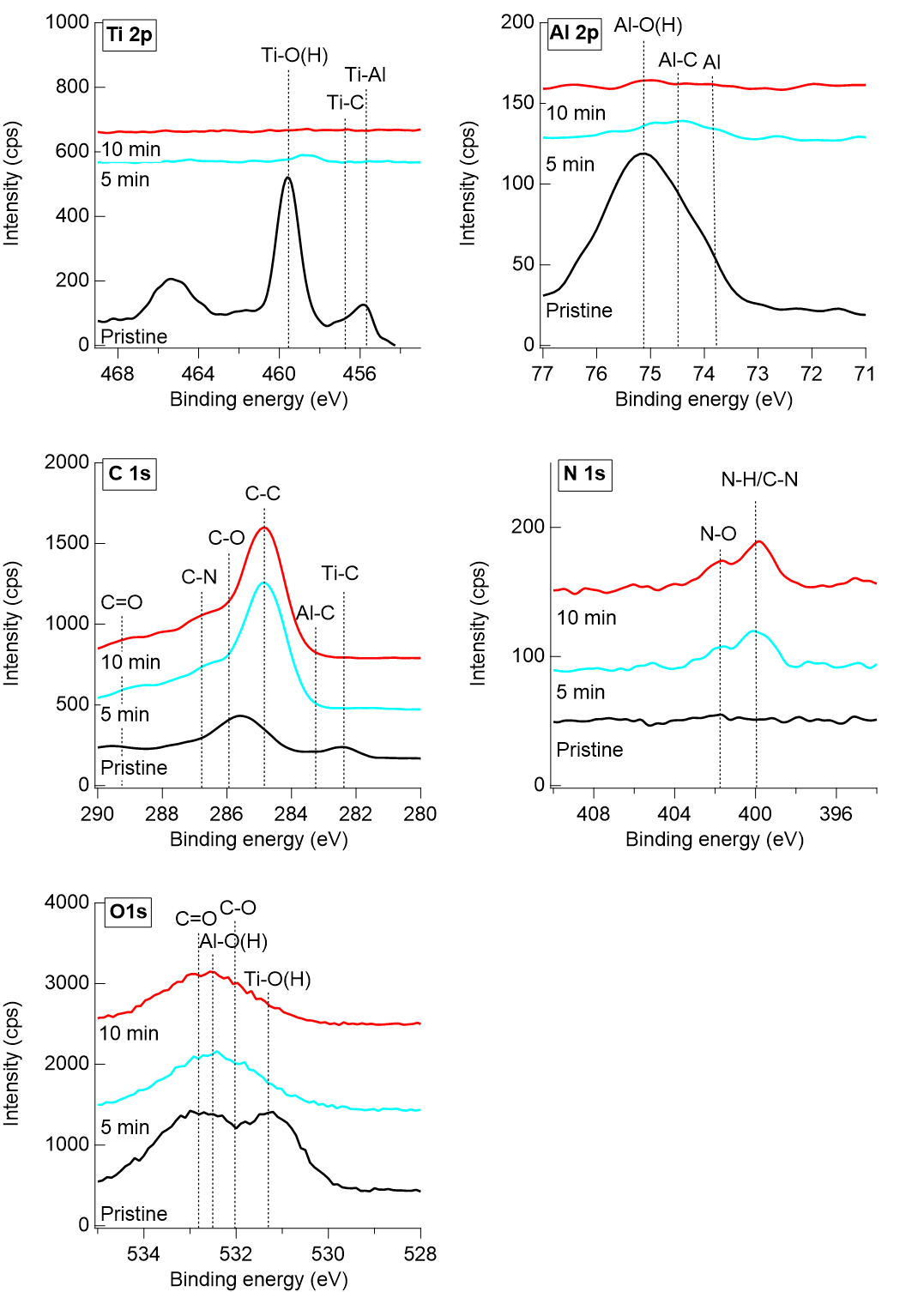 |
| --- |

Figure S2. XPS spectra obtained on the surface of TiAlC film after wet chemical etching in NH_4_OH/H_2_O_2_/H_2_O solution that was mixed at room temperature. Etch time was used as a parameter in the range from 0 to 10 min.

Table S1. Film thickness of TiAlC film and the surface layer etched by NH_4_OH/H_2_O_2_/H_2_O (2.2:3:52) mixture (condition L4). Film thickness was evaluated by using ellipsometry.

| Etch time (min) | Pristine TiAlC | | | After (NH_4_OH, H_2_O_2_, H_2_O) wet etching | | | |
| --- | --- | --- | --- | --- | --- | --- | --- |
|  | Film thickness (native oxide) | Film thickness (TiAlC) (nm) | Film thickness (top layer) (nm) | | Film thickness (TiAlC) (nm) | Etch rate-total film (nm/min) | Etch rate-TiAlC (nm/min) |
| 5 | 3.7 | 32.7 | 13.3 | | 8.1 | 3.0 | 4.9 |
| 10 | 3.8 | 32.6 | 11.7 | | 3.0 | 2.3 | 3.1 |
| 15 | 3.8 | 33.2 | 7.4 | | 2.4 | 1.8 | 2.1 |
| 20 | 3.8 | 33.0 | 7.1 | | 2.2 | Etch stop | Etch stop |
